# Tendon-Specific Dicer Deficient Mice Exhibit Hypoplastic Tendon Through the Downregulation of Tendon-Related Genes and MicroRNAs

**DOI:** 10.3389/fcell.2022.898428

**Published:** 2022-06-14

**Authors:** Takenori Omoto, Dilimulati Yimiti, Yohei Sanada, Minoru Toriyama, Chenyang Ding, Yuta Hayashi, Yasunari Ikuta, Tomoyuki Nakasa, Masakazu Ishikawa, Masayuki Sano, Minjung Lee, Takayuki Akimoto, Chisa Shukunami, Shigeru Miyaki, Nobuo Adachi

**Affiliations:** ^1^ Department of Orthopaedic Surgery, Graduate School of Biomedical and Health Sciences, Hiroshima University, Hiroshima, Japan; ^2^ Medical Center for Translational and Clinical Research, Hiroshima University Hospital, Hiroshima, Japan; ^3^ Department of Musculoskeletal Traumatology and Reconstructive Surgery, Graduate School of Biomedical and Health Sciences, Hiroshima University, Hiroshima, Japan; ^4^ Department of Artificial Joints and Biomaterials, Graduate School of Biomedical and Health Sciences, Hiroshima University, Hiroshima, Japan; ^5^ Cellular and Molecular Biotechnology Research Institute, National Institute of Advanced Industrial Science and Technology, Tsukuba, Japan; ^6^ Faculty of Sport Sciences, Waseda University, Saitama, Japan; ^7^ Department of Molecular Biology and Biochemistry, Graduate School of Biomedical and Health Sciences, Hiroshima University, Hiroshima, Japan

**Keywords:** tendon, dicer, microRNA, knockout mice, extracellular matrix, collagen fibrils, tendon abnormality

## Abstract

Tendon is a fibrous connective tissue, that is, transmitting the forces that permit body movement. However, tendon/ligament biology is still not fully understood and especially, the role of miRNAs in tendon/ligament is sparse and uncharacterized in *in vivo* models. The objectives of this study were to address the function of DICER using mice with tendon/ligament-specific deletion of *Dicer* (*Dicer* conditional knockout; cKO), and to identify key miRNAs in tendon/ligament. *Dicer* cKO mice exhibited hypoplastic tendons through structurally abnormal collagen fibrils with downregulation of tendon-related genes. The fragility of tendon did not significantly affect the tensile strength of tendon in *Dicer* cKO mice, but they showed larger dorsiflexion angle in gait compared with Control mice. We identified two miRNAs, miR-135a and miR-1247, which were highly expressed in the Achilles tendon of Control mice and were downregulated in the Achilles tendon of *Dicer* cKO mice compared with Control mice. miR-135a mimic increased the expression of tendon-related genes in injured Achilles tendon-derived fibroblasts. In this study, *Dicer* cKO mice exhibited immature tendons in which collagen fibrils have small diameter with the downregulation of tendon-related genes such as transcriptional factor, extracellular matrix, and miRNAs. Thus, DICER plays an important role in tendon maturation, and miR-135a may have the potential to become key miRNA for tendon maturation and healing.

## Introduction

Tendon and ligament are fibrous connective tissues consisting mainly of collagen that are critical parts of the musculoskeletal system. They transmit the forces that permit smooth body movement. Although tendon injury is a major clinical problem in the field of sports and aging, tendon healing such as in Achilles tendon rupture is very slow and frequently demonstrate recovery with ossification and scar formation (Ateschrang et al., 2007; Huttunen et al., 2014). Thus, further new interventions are required to accelerate tendon healing without ossification and scar formation based on the molecular mechanisms of development and homeostasis of tendon.

In tendon biology, tendon/ligament development including tenogenic/ligamentagenic lineage is not yet fully characterized because of the absence of tendon/ligament-specific marker genes. Furthermore, the lack of tools such as ligament/tendon-specific Cre driver mouse is one of the causes of limited understanding of tendon/ligament biology. However, it has recently been demonstrated that several key transcriptional factors and extracellular matrix (ECM) are involved in the development and maturation of tendon/ligament from transgenic mouse model (Delgado Caceres et al., 2018). Transcriptional factors such *Scleraxis* (*Scx*), *Mohawk* (*Mkx*) and *EGR1* are critical regulators of tendon differentiation and maturation, and they also regulate tendon/ligament matrix genes such as *Col1a1* and *Tenomodulin* (*Tnmd*) (Murchison et al., 2007; Ito et al., 2010; Guerquin et al., 2013; Yoshimoto et al., 2017). It has been known that *Scx* is expressed in tendon fibroblasts from various previous studies using transgenic reporter mice (Pryce et al., 2007; Sugimoto et al., 2013; Yoshimoto et al., 2017). As a suitable Cre driver mouse for ligament/tendon study, the *ScxCre* knock-in (KI) mouse line has been recently generated and is able to inactivate endogenous *Scx* by driving *Cre* expression using the endogenous *Scx* promoter (Yoshimoto et al., 2017).

MicroRNAs (miRNAs) are a class of noncoding RNAs that negatively regulate the expression of genes through binding to complementary target mRNAs. Primary transcripts (pri-miRNAs) and precursor miRNA (pre-miRNA) molecules undergo nuclear and cytoplasmic processing events, carried out by the endoribonucleases, DROSHA and DICER, respectively, to produce mature miRNAs that are loaded onto the RISC (RNA-induced silencing complex) to exert their biological function (Lee et al., 2003; Michlewski and Cáceres, 2018). Limb- or cartilage-specific *Dicer*-deficient mice exhibit a severe phenotype in skeletal development (Harfe et al., 2005; Kobayashi et al., 2008). Deletion of *Drosha* or *DGCR8* in chondrocytes causes a lethal skeletal defect like that of *Dicer* deletion, confirming the essential role of miRNAs in normal skeletogenesis (Kobayashi et al., 2015). Mature miRNAs play an important role in various developmental and disease processes in the musculoskeletal system. For example, we previously reported that the cartilage-specific miRNA, miR-140, regulates cartilage development and homeostasis, and that its loss contributes to the development of age-related osteoarthritis in deficient mice (Miyaki et al., 2009; Miyaki et al., 2010). These observations suggest that DICER and miRNAs could contribute to the development of the tendon/ligament. Although it has been reported that several miRNAs are associated with tendon development and homeostasis modulation (Dubin et al., 2018), the roles of DICER and miRNAs in the development and homeostasis of tendon/ligament have not been well-characterized due to the lack of *in vivo* studies using transgenic mice.

The objective of this study was to establish the roles of DICER-miRNAs in tendon/ligament development using tendon/ligament-specific *Dicer* deficient mice.

## Materials and Methods

### Generation of Tendon/Ligament-Specific *Dicer* Deficient Mice

Ligament/tendon-specific *Dicer* deficient (*Dicer* cKO) mice were generated by crossbreeding the previously described *Dicer* 1-floxed mice (Harfe et al., 2005) and *ScxCre* KI mice (Yoshimoto et al., 2017). The conditional floxed allele and the deletion allele were genotyped using primers as previously described (Harfe et al., 2005; Yoshimoto et al., 2017) (Supplementary Figure S1A). All mice were housed in temperature-controlled quarters (23 ± 1°C) with a 12-h light-dark cycle and in groups of two to five per cage (S cage: 143 mm × 293 mm × H148 mm) and provided with free access to water and food. All animal experiments were performed according to protocols approved by the Hiroshima University Animal Care and Use Committee.

### Macroscopic and Histological Analysis

After removing the skin, various tendons were macroscopically observed in mice of three genotypes. Hind limbs including Achilles tendon were embedded intact in paraffin after fixation with 4% paraformaldehyde phosphate buffer solution (PBS) for 48 h and decalcification in 18.5% EDT-X (Falma, Tokyo, Japan) for 3 weeks. Tissue sections were sectioned (4.5 μm) in the sagittal or transverse plane. The sections were stained with Hematoxylin and Eosin (MUTO PURE CHEMICALS, Tokyo, Japan), Safranin-O (MUTO PURE CHEMICALS, Tokyo, Japan)/Fast green (Sigma-Aldrich, United States), and Picro-Sirius Red stain kit (ScyTek labolatories inc.). We also measured the transverse-section area of the center of Achilles tendon. Analysis of the transverse section area was carried out using ImageJ software.

### Tendon Injury Model and Histological Analysis

In tendon injury model, the left Achilles tendon in Control and *Dicer* cKO mice at 10 weeks of age was exposed after longitudinal skin incision. Without attempt at repair, a complete transverse incision was made at midpoint of the Achilles tendon with scissors (Asai et al., 2014). Four weeks after injury, the histologic tendon healing was evaluated using modified tendon healing scoring and chondrification scoring system (Hayashi et al., 2022) that excluded some items from the previous study (Stoll et al., 2011).

### Transmission Electron Microscopy

Hind limbs were dissected from mice at 10 weeks of age and were fixed in 2.5% glutaraldehyde (FUJIFILM WAKO) in 0.1 M sodium cacodylate buffer (FUJIFILM WAKO) for 24 h at 4°C. After 24 h, approximately 1 mm^3^ of small pieces that were isolated from the central region of repaired Achilles tendon tissues were fixed in 2.5% glutaraldehyde (FUJIFILM WAKO) in 0.1 M sodium cacodylate buffer (FUJIFILM WAKO) at 4°C for 24 h additionally. Samples were then dehydrated using a graded ethanol series and embedded in EPOK 812 resin (Okenshoji, Japan) using a graded resin and propylene oxide series. Ultrathin sections with silver-gold reflectance were obtained with an ultramicrotome (ULTRACUT E, Reichert-Jung, Austria) and stained with 3% uranyl acetate and lead citrate, then observed using a JEM-1400 (JEOL, Japan) TEM operating at an accelerating voltage of 80 kV. Fibril diameter analyses were done from transverse section images. All fibrils within a predetermined region of interest (ROI) on the digitized image were measured using ImageJ, as described (Starborg et al., 2013).

### Edu Staining

Edu staining was performed using Click-iT Plus EdU Alexa Fluor™ 488 Imaging kit (Thermo Fisher Scientific). Nuclei were stained by Hechst 33324 (Thermo Fisher Scientific) and Edu-positive cells were measured in Achilles tendons. Results are expressed as percentage of Edu-positive cells relative to the total number of tendon fibroblasts.

### Immunohistochemistry

The deparaffinized paraffin sections were revitalized with antigen-retrieval reagent (Immunoactive; Matsunami Glass Ind, Osaka, Japan) at 60°C for 16 h or by Proteinase K (Dako) and blocked in 5% goat serum with 1% bovine serum albumin (BSA, FUJIFILM WAKO). After blocking, the samples were incubated with anti-TENOMODULIN antibody (1:200; ab203676, Abcam), anti-PRO COLLAGEN TYPE I antibody (DSHB, SP1.D8, 1:100), and anti-COLLAGEN TYPE II antibody (DSHB, CII C1, 1:500) overnight at 4°C. The sections were then incubated with Alexa Fluor 568 goat anti-rabbit antibody (1:500; Thermo Fisher Scientific) or Alexa Fluor 568 goat anti-mouse antibody (1:500; Thermo Fisher Scientific) for 1 h. Nuclei were stained using 4′,6-diamidino-2-phenylindole (DAPI) (DOJINDO, Japan).

### Grip Strength and Mechanical Properties of Tensile Strength

Grip strength of the forelimbs was measured using Grip strength meter (MK-380Si; Muromachi Kikai Co., Ltd.). In biomechanical testing of Achilles tendons, a uniaxial material testing system (EZ-SX; SHIMADZU, Japan) was used to determine tensile strength with a 500 N load. The proximal end of the Achilles tendon and foot of the mouse were fixed in clamps with wet kimtowel and the specimen was tensiled at a fixed strain rate of 5 mm/min. The entire tendon unit from myotendinous junction to the calcaneus was investigated. Breaking strength and elongation were measured, and tensile strength per unit area was calculated based on the average of cross section area of Achilles tendon at 10 weeks of age.

### Gait Analysis Using a New System With Artificial Intelligence

Mice walked on a treadmill MK 690S04 MDH (Muromachi Kikai Co., Ltd.) at 10 m/min and video was captured from the sagittal plane at 30 fps using a smartphone. Gait analysis was performed using a new system with AI, Deep Treated M1 (Research Coordinate, Inc., Tokyo). The computer, that previously learned the movements of optional parts of the subject on the video using neural net models with deep learning, performed auto tracking of the subject’s lower limb landmarks and quantified spatial position data along the timeline. Landmarks horizontal position of the video was converted into the X-axis data and the vertical position into the Y-axis. In this study, knee joint, heel and fifth metatarsal head position values were obtained, and ankle angle was calculated using the inverse function on the obtained values (Supplementary Video 1). We subtracted 90° from the obtained ankle joint angle and defined the results of positive value as a plantar-flexion angle and a negative value as a dorsiflexion angle. The maximum dorsi-flexion and plantar-flexion angle in one gait cycle were obtained, and angular velocity through the entire gait cycle was calculated respectively. The sum of maximum dorsi-flexion and plantar-flexion was defined as range of motion (ROM), and coefficient of variation (CV) for the period of gait cycles through the measured time was calculated. All data were processed in a program designed with MATLAB (MathWorks, Natick, MA).

### RNA Isolation and RT-PCR of Tendon Tissue

The total RNA was extracted from Achilles tendon in mice and cultured tendon fibroblasts using Isogen reagent (Nippon gene, Tokyo, Japan) and RNA purification kit (Direct-zol RNA microprep, Zymo Research, California, United States). Complementary DNA (cDNA) was synthesized with a Reverse Transcription system (iScript supermix, BioRad, California, United States) according to the manufacturer’s protocol. Real-time polymerase chain reaction (real-time PCR) was performed with the TaqMan Gene Expression Assay probes (Thermo Fisher Scientific) for tendon-related markers (Supplementary Table S1). Real-time PCR for miRNAs (Supplementary Table S1) was performed using the TaqMan MicroRNA Reverse Transcription kit (Thermo Fisher Scientific). *Gapdh* (Mm99999915g1) or U6 snRNA (RT/TM001973) were used as internal controls to normalize sample differences. The ΔΔCt method was used for analysis of real-time PCR data.

### Isolation of Tendon Fibroblasts

Healing Achilles tendons were taken out from C57BL6/J mice 1 week after injury and the tendon healing portion tissues were then minced. Tissue sample was digested with 3 mg collagenase type I (Worthington Biochemical Co., Lakewood, NJ) and 1 mg dispase (FUJIFILM WAKO) in Hanks’ Balanced Salt Solution (HBSS; FUJIFILM WAKO) at 37°C for 1 h with gentle shaking (Bi et al., 2007). Tendon fibroblasts were cultured in Minimum Essential Medium Eagle Alpha Modification (MEMα; FUJIFILM WAKO) with 20% fetal bovine serum (FBS; Thermo Fisher Scientific) and 1% Penicilin-Streptomycin-Amphotericin B Suspension (FUJIFILM WAKO) at 37°C with 5% CO2. To introduce miRNA mimic, injured Achilles tendon-derived fibroblasts (iATDF) were transfected with 30 nM of double-stranded miRNA for miR-135a: (sense) 5′ UAU​GGC​UUU​UUA​UUC​CUA​UGU​GA-3′ and (antisense) 5′-UAU​AGG​GAU​UGG​AGC​CGU​GGC-3′, or miR-1247: (sense) 5′ ACC​CGU​CCC​GUU​CGU​CCC​CGG​A-3′ and (antisense) 5′-CGG​GAA​CGU​CGA​GAC​UGG​AGC-3’ (Hokkaido System Sciences, Hokkaido Japan), using Lipofectamine RNAi Max Reagent (Invitrogen, Carlsbad, CA, United States) according to the manufacturer’s instructions. Control siRNAs were also prepared for the control group (siRNA negative control; siNega #1, Invitrogen). Forty-eight hours after transfection, iATDF were washed with PBS and cultured with MEMα/10% FBS for an additional 7 days. All experiments used tendon fibroblasts at passage one to two.

### Statistical Analysis

Data are plotted as individual points with bars indicating mean standard deviation (SD) or standard error of the mean (SEM). Statistically significant differences between the two groups or three were determined with Welch’s *t* test, Mann-Whitney U test, and One-way ANOVA and LSD test. Differences were considered statistically significant at *p* < 0.05.

## Results

### Generation of Tendon-Specific *Dicer* Deficient Mice

**FIGURE 1:**
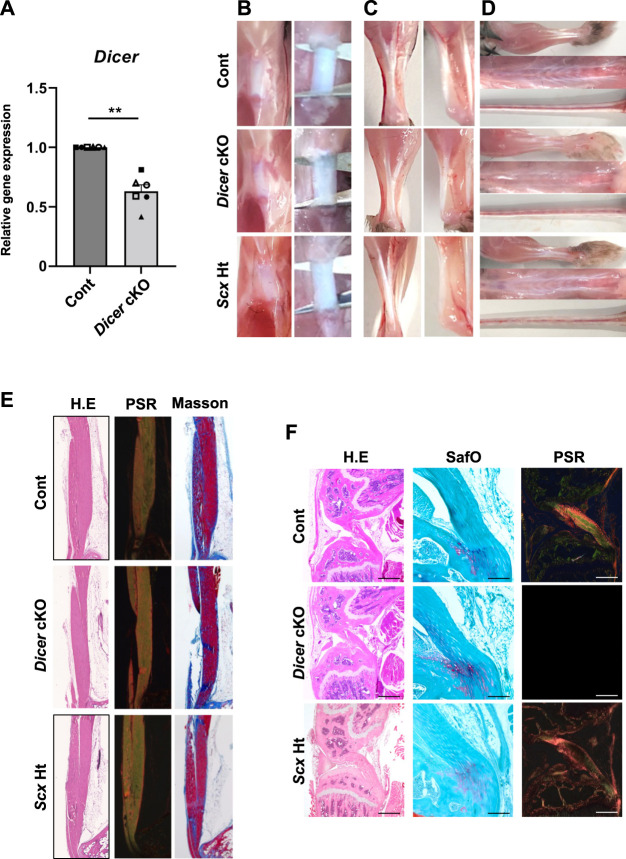
Macroscopic and histological finding in Tendons of Dicer cKO mice. **(A)** The expression of *Dicer* in Achilles tendon of Cont and *Dicer* cKO mice (*n* = 6 mice for each genotype) at 4 weeks of age was shown by real-time PCR. Data are represented as mean ± SEM. Comparison of *Dicer* expression was performed using Mann-Whitney U test; **p < 0.01. **(B)** Macro view of Patellar tendon, **(C)** Achilles tendon, **(D)** forelimb tendons, back tendon and tail tendons in *Dicer* ^f/f^ (Cont), *Scx* ^Cre/+^KI:*Dicer* ^flox/flox^ (*Dicer* cKO), and *Scx* ^Cre/+^KI:*Dicer* ^+/+^ (*Scx*Ht) mice at 10 weeks of age. **(E)** Hematoxylin and Eosin (H.E), Picsirius red and Masson trichrome staining of Achilles tendon of Cont, *Dicer* cKO, and *Scx*Ht mice at 10 weeks of age. **(F)** H.E, safranin O/Fast green and Picsirius red staining of cruciate ligament of Cont, *Dicer* cKO, and *Scx*Ht mice at 10 weeks of age. Scale bars: 100 and 500 µm.

To examine the physiological roles of *Dicer* in tendon/ligament development, we generated tendon/ligament-specific *Dicer* deficient mice (Supplementary Figure S1A). *ScxCre*-mediated excision is mainly observed in domains with persistent *Scx* expression, such as tendons and ligaments (Yoshimoto et al., 2017). *Dicer* was significantly downregulated in the Achilles tendons of *Dicer* cKO compared with those of Control mice at 4 weeks of age (Figure 1A). The appearance, including body length and weight, was not significantly different among *Scx* ^Cre/+^ KI:*Dicer* ^f/f^ (*Dicer* cKO) mice, their littermate controls; *Dicer* ^f/f^ (Control) mice and *Scx* ^Cre/+^ KI:*Dicer* ^+/+^ (*Scx*Ht) mice at 10 weeks of age (Supplementary Figures S1B,C).

### Impaired Maturation, Healing, and Function of Achilles Tendon in *Dicer* Conditional Knockout Mice

**FIGURE 2:**
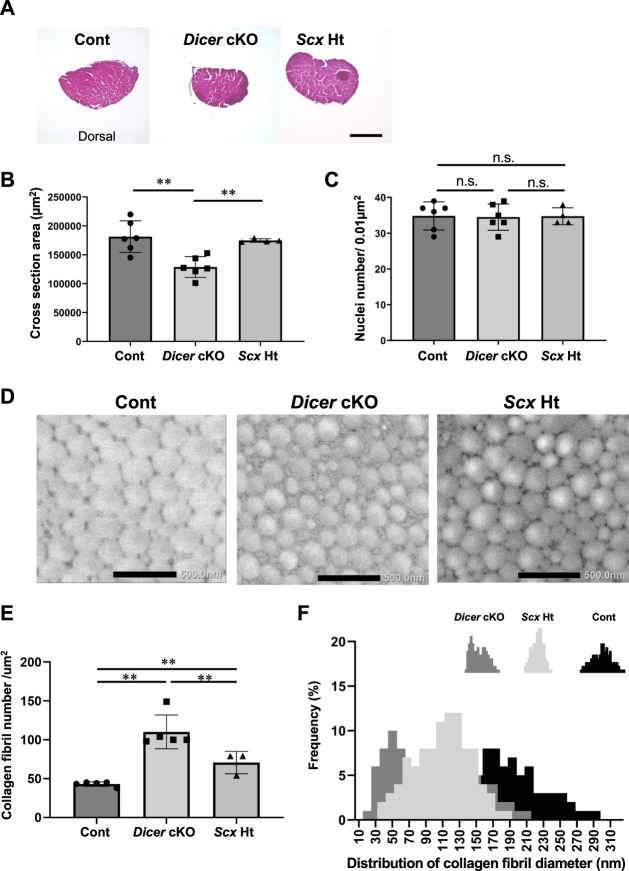
Abnormal structure in Achilles tendon of *Dicer* cKO mice. **(A)** Hematoxylin and Eosin (H.E) staining of cross section of Achilles tendon in Cont, *Dicer* cKO, and *Scx*Ht mice at 4 weeks of age. **(B)** Cross section area (µm^2^) and **(C)** the number of nuclei per unit area of a section of Achilles tendon at 4 weeks of age (*n* = 4–6 mice for each genotype). **(D)** Transmission electron microscopic view of collagen fibrils in Achilles tendon of Cont, *Dicer* cKO and *Scx*Ht mice at 10 weeks of age (*n* = 3–5 mice for each genotype). Scale bars: 500 nm. **(E)** Collagen fibril number per µm^2^ was significantly increased in *Dicer* cKO mice compared with Cont and *Scx*Ht mice. **(F)** Distribution of collagen fibril diameter shifted to smaller diameter fibrils in *Dicer* cKO mice. The data are represented as mean ± S.D. Comparison of mean values was performed using One-way ANOVA and Fisher’s LSD test; **p < 0.01. n.s.: no significant difference.

To investigate the role of DICER in tendon development and maturation, we performed a macroscopic, a histological, and an ultrastructural analysis of the Control, *Scx*Ht and *Dicer* cKO mice. Patellar tendon, Achilles tendons, the forelimb tendon, back tendons and tail tendon, exhibited gross fragility in *Dicer* cKO mice compared with those of Control and *Scx*Ht mice (Figures 1B–D). Histological analysis of the sagittal section did not find remarkable histological changes to the patellar tendon, Achilles tendon and cruciate ligament in *Dicer* cKO mice (Figures 1E,F). Fibrocartilaginous entheses were histologically normal (Figure 1F). However, in a quantitative analysis of Achilles tendon using transverse section, the area of the Achilles tendons was significantly smaller in *Dicer* cKO mice compared with Control and *Scx*Ht mice at 4 weeks of age (Figures 2A,B). The number of cells per unit area of the section of Achilles tendons was similar between Control, *Scx*Ht and *Dicer* cKO mice (Figure 2C).

**FIGURE 3:**
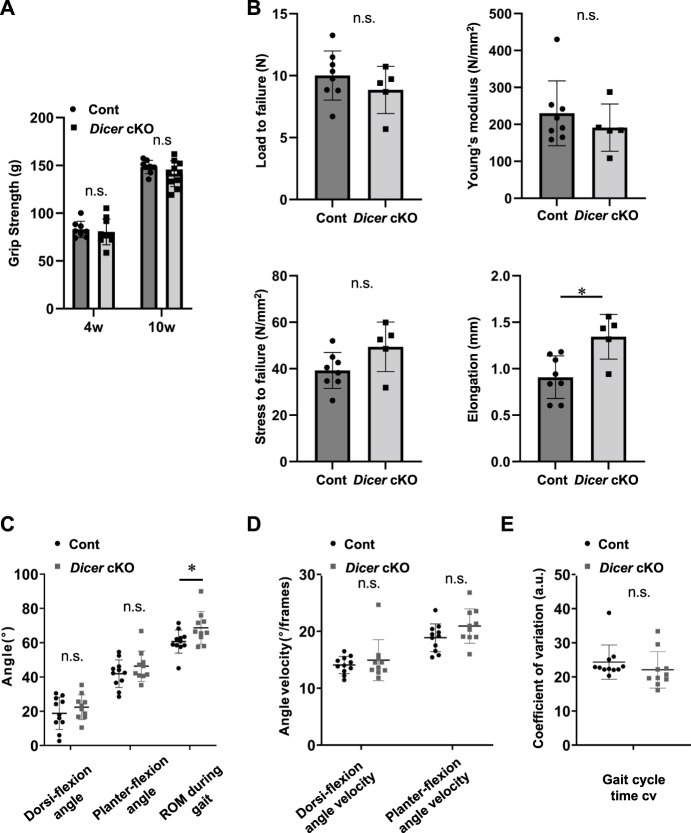
Function of tendon in *Dicer* cKO mice. **(A)** Grip strength of forelimb in Cont (*n* = 8) and *Dicer* cKO (*n* = 10) mice at 4 and 10 weeks of age. **(B)** Load and stress to failure in Achilles tendons of Cont (*n* = 8) and *Dicer* cKO (*n* = 5) mice at 10 weeks of age. **(C)** Gait analysis was performed using Cont (*n* = 11) and *Dicer* cKO (*n* = 10) mice at 10 weeks of age. Maximum dorsi-flexion angle, plantar flexion angle, ROM during gait **(C)**, dorsi-flexion angular velocity, plantar flexion angular velocity **(D)**, and gait cycle time CV **(E)**. Data are represented as mean ± S.D. Comparison of mean values was performed using **(A)** Welch’s *t* test with Holm-Sidak correction for multiple comparison, and **(B–E)** Welch’s *t* test; ^*^ *p* < 0.05 versus Cont. n.s.: no significant difference.

To further examine the tendons of *Dicer* cKO mice in detail, we performed ultrastructural analysis of Achilles tendon using TEM. Collagen fibrils of *Dicer* cKO mice exhibited structurally abnormal fibrils with irregular fibril profiles including many fibrils with small diameter compared with that of Control and *Scx*Ht mice at 10 weeks of age (Figures 2D–F). Although tendon development of ScxHt mice was grossly normal as previously reported (Yoshimoto et al., 2017), ultrastructural analysis by TEM showed significant difference between *Dicer* cKO and ScxHt mice. To examine the functional abilities of Achilles tendons in Control and *Dicer* cKO mice, we performed grip strength testing, biomechanical testing and a gait analysis. The grip strength of forelimb was not significantly different between *Dicer* cKO and Control mice at 4 and 10 weeks of age (Figure 3A). Tensile strength of the Achilles tendon (represented by load to failure and stress to failure) was similar between both type of mouse (Figure 3B). However, the elongation of the Achilles tendons was significantly increased in *Dicer* cKO mice (Figure 3B, Supplementary Video 2). The dorsiflexion angle, plantar flexion angle and range of motion (ROM) were analyzed to examine ankle motion during gait. ROM was higher in *Dicer* cKO mice than in Control mice (Figure 3C). Both plantar flexion and dorsiflexion angular velocity and gait cycle time coefficient of variation (CV) were not significantly different between Control and *Dicer* cKO mice (Figures 3D,E).

**FIGURE 4:**
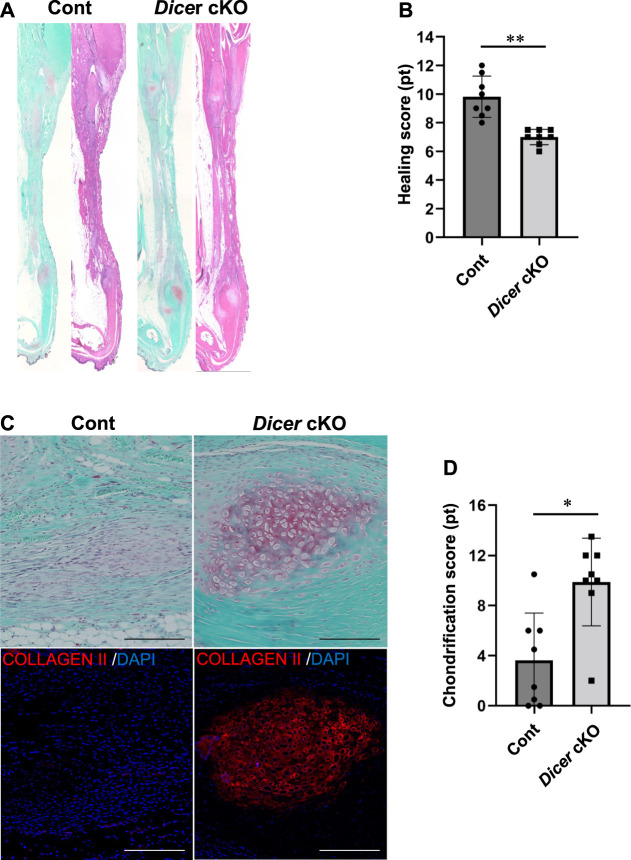
Tendon healing in *Dicer* cKO mice. **(A)** Hematoxylin and Eosin (HE) staining of Achilles tendons of Cont and *Dicer* cKO mice at 4 weeks after injury. **(B)** Tendon healing scores were significantly decreased in *Dicer* cKO mice compared with Cont mice. **(C)** Chondrification parts with Safranin O- and type II COLLAGEN-positive were more frequently observed in *Dicer* cKO mice during healing. Scale bars: 200 µm. **(D)** Chondrification scores were significantly increased in *Dicer* cKO mice compared with Cont mice. *n* = 8 mice for each genotype. Data are represented as mean ± S.D. Comparison of mean values was performed using Welch’s *t* test; *p < 0.05 and **p < 0.01 versus Cont. n.s.: no significant difference.

We further investigated the involvement of DICER in tendon healing using tendon injury model. At 4 weeks after injury, histological analysis using tendon healing scoring indicated a significant decrease in the healing capacity of the Achilles tendons (Figures 4A,B). Tendon ossification *via* chondrification, which causes clinical problems following tendon healing, is often observed in sites of tendon healing. Achilles tendons exhibiting chondrification parts with Safranin O- and type II COLLAGEN-positive were observed in Control and *Dicer* cKO mice. However, ectopic chondrification during tendon healing was significantly increased in *Dicer* cKO mice compared with Control mice (Figures 4A,C,D). These results suggest that *Dicer* is positively involved in tendon healing too.

### Proliferating Cells in Achilles Tendon of *Dicer* Conditional Knockout Mice

**FIGURE 5:**
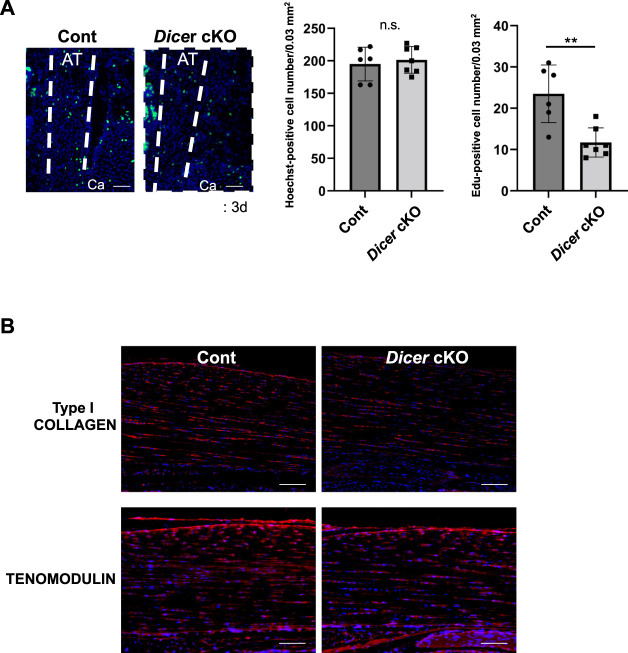
Cellularity in Achilles tendons of *Dicer* cKO mice. **(A)** Edu-positive cells were decreased in Achilles tendons of *Dicer* cKO mice at postnatal day 3 (*n* = 6–7 mice for each genotype). Scale bars: 50 µm. **(B)** Immunohistochemistry using anti-TENOMODULIN antibodies and anti-PRO COLLAGEN TYPE I antibodies of Achilles tendons of Cont and *Dicer* cKO mice at 3 weeks of age (*n* = 5 per group). Data are represented as mean ± S.D. Comparison of mean values was performed using **(A)** Welch’s *t* test and **(B)** two-way ANOVA; *p < 0.05, **p < 0.01 versus Cont. AT: Achilles tendon. Ca: calcaneus. Scale bars: 100 μm. n.s.: no significant difference.

DICER is involved in cell death, proliferation, and differentiation (Guo and Wang, 2019). The number of EdU-positive tendon fibroblasts was significantly reduced in the Achilles tendons of *Dicer* cKO mice compared with Control mice at postnatal day 3 (Figure 5A). On the other hand, TUNEL-positive tendon fibroblasts were undetected in Achilles tendon of *Dicer* cKO mice at postnatal day 3 (data not shown). In immunohistochemistry, the differences of type I COLLAGEN- and TENOMODULIN**-**positive cells were not clearly detected in Achilles tendon between Cont and *Dicer* cKO mice (Figure 5B).

### Expression Pattern of Tendon-Related Genes and MicroRNAs in Achilles Tendon of *Dicer* Conditional Knockout Mice

**FIGURE 6:**
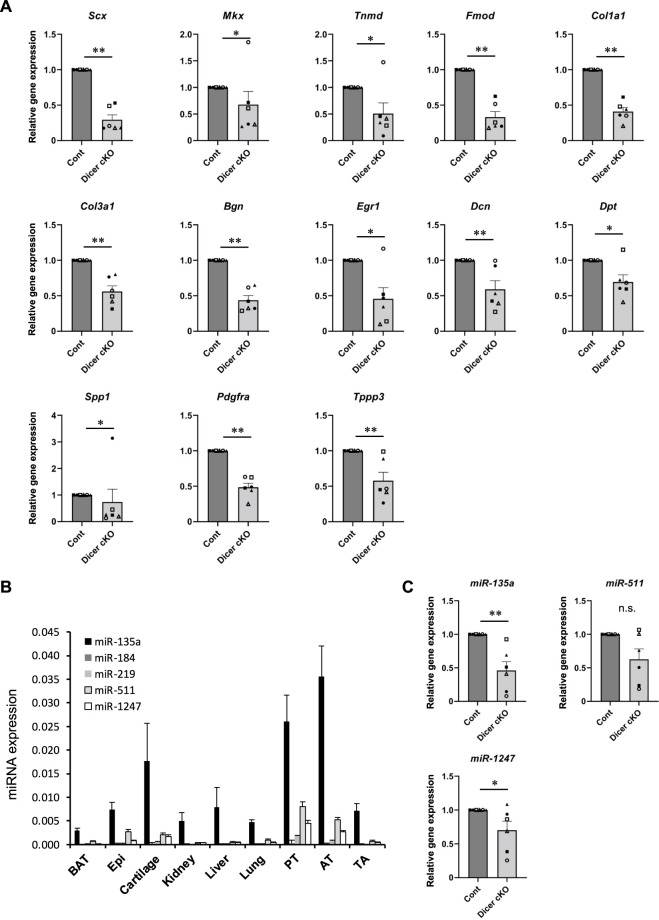
Tendon related-genes and -miRNAs expression in Achilles tendon tissues of *Dicer* cKO mice. **(A)** The expression of tendon-related genes in Achilles tendon tissues from Cont and *Dicer* cKO mice (*n* = 6 each group) at 4 weeks of age. **(B)** The expression of selected miRNAs in various tissues from C57BL6/J mice (*n* = 4). **(C)** The expression of tendon-related miRNAs in Achilles tendon tissues from Cont and *Dicer* cKO mice (*n* = 6 mice for each genotype) at 4 weeks of age. All data represented as mean ± SEM. Comparison of genes or micro RNAs expression was performed using Mann-Whitney U test; ^*^ *p* < 0.05, and **p < 0.01 versus Cont. n.s.: no significant difference.

**FIGURE 7:**
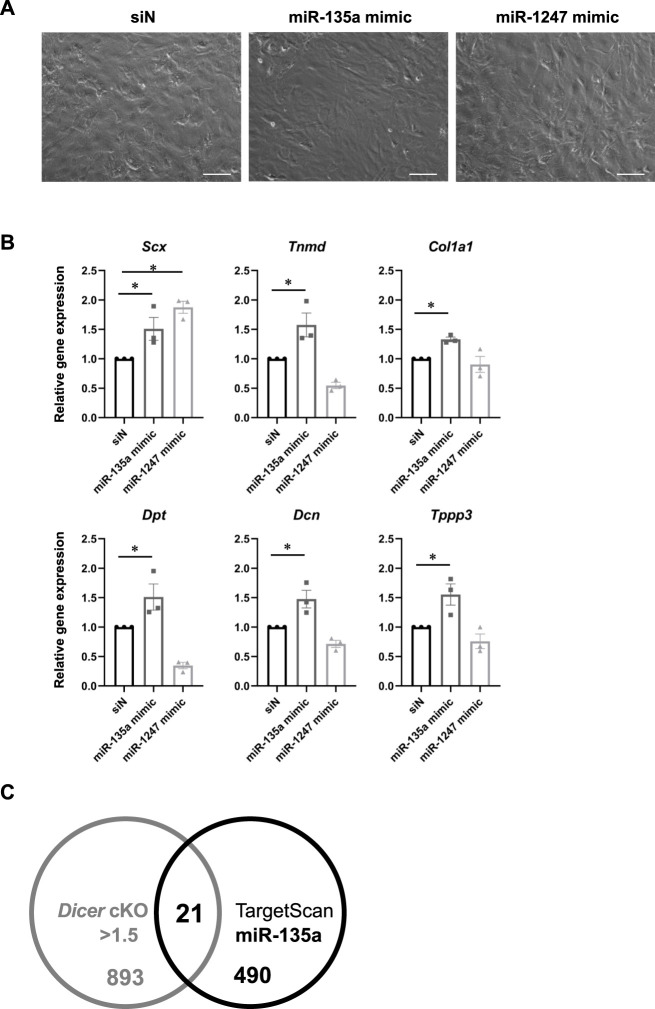
Tendon related-genes expression in injured Achilles tendon-derived fibroblasts with miR-135a mimic. **(A)** Cell morphology in cultured injured Achilles tendon-derived fibroblasts (iATDF) with miR-135a mimic and miR-1247 mimic (Final 30 nM). **(B)** The expression of tendon-related genes in iATDF with miR-135a and miR-1247 mimic (*n* = 3). **(C)** Venn diagram comparing upregulated genes in *Dicer* cKO (*Dicer* cKO/Control ratio >1.5, baseMean >50 counts) and candidate genes of miR-135a in RNA-seq analysis and TargetScan. The data represented as mean ± SEM. Comparison of genes expression was performed using Kruskal-Wallis test; ^*^ *p* < 0.05 versus siN (Control). siN: siRNA negative control. Scale bars: 100 µm.

To investigate which mRNA and miRNA are involved in *Dicer* cKO mouse tendon hypoplasticity, we performed RNA-sequencing (RNA-seq) and small RNA-sequencing (miRNA-seq) using RNA from Achilles tendon of Control, *Scx*Ht and *Dicer* cKO mice at 4 weeks of age. Although the diversity of cell types that populate the tendon has not been understood in detail yet, recently, extensive heterogeneity of the cellular composition of mouse’ Achilles tendons has been identified using single-cell transcriptomic analysis (De Micheli et al., 2020). Based on this data set, we focused on the expressing gene sets (tendon fibroblasts-related genes) in cell type that were classified as tendon fibroblast. RNA-seq analysis showed that most tendon fibroblasts-related genes were decreased in *Dicer* cKO mice compared with Control mice (Supplementary Figures S2A, 3 and Supplementary File). To further investigate the altered biological processes as a result of *Dicer* cKO, gene ontology (GO) analysis was performed. The upregulated genes were involved in muscle system process and mitotic cell cycle, and downregulated genes were involved in ossification, skeletal system development, and extracellular matrix organization (Supplementary Figure S2B). We validated the expression of tendon-related genes in Achilles tendon by real-time PCR. Tendon lineage-related transcriptional factors, tendon matrix genes, ECM binding genes, and tendon stem/progenitor cells (TSPCs) markers (Huang et al., 2021), tublin polymerization-promoting protein family member 3 (*Tppp3*) and platelet-derived growth factor receptor alpha (*Pdgfra*) were significantly decreased in Achilles tendon of *Dicer* cKO mice at 4 weeks of age compared with Control mice (Figure 6A). Furthermore, the reduction of expressed miRNAs in tendon could be associated with the hypoplastic changes to the tendons of *Dicer* cKO mice. We, therefore, examined which specific miRNAs were involved. We identified differentially expressed top 10 miRNAs which were downregulated in the Achilles tendons of *Dicer* cKO mice compared with Control mice (cut off: *Dicer* cKO/Control ratio >0.6, baseMean >15 counts) (Supplementary Figure S4 and Supplementary Table S2). Among them, miR-135a, miR-511 and miR-1247 had specific expression patterns in tendons compared with various tissues in C57BL6/J mice at 8 weeks of age (Figure 6B). The expression of miR-135a and miR-1247, which was validated by real-time PCR, was found to be significantly downregulated in the Achilles tendons of *Dicer* cKO mice (Figure 6C). Finally, the introduction of miR-135a mimic into iATDF increased the expression of tendon-related genes such as *Tnmd* and *Tppp3* (Figures 7A,B), but not with the introduction of miR-1247. Identification of miR-135a target genes might provide an insight into the mechanism of phenotype of *Dicer* cKO mice. Based on predicted target genes for miR-135a found in the database, TargetScan 7.2 (https://www.targetscan.org/vert_72/docs/help.html), 21 upregulated genes in Achilles tendon of *Dicer* cKO mice were listed (cut off: *Dicer* cKO/Control ratio >1.5, baseMean >50 counts. Figure 7C and Table 1).

**TABLE 1:** Target candidate genes for miR-135a.

Gene symbol	Base mean	Fold change
Cadm4	74.2396	3.3231
Arhgap19	133.0803	2.5688
Adcyap1r1	187.4673	2.4734
Eya1	157.7262	2.4566
Csmd1	67.4648	2.1977
Grid2	50.3297	2.1084
Tefm	93.5072	1.8444
Elovl6	732.3945	1.8217
Slc24a2	142.1385	1.6577
Atg14	169.2565	1.6536
Pcyt1b	63.7849	1.6531
Cplx2	93.4129	1.6442
Ccng2	264.1414	1.6261
Lrrn1	546.8143	1.6100
Acvr1b	238.0046	1.6079
Zdhhc23	111.4538	1.5813
Tsen54	52.7075	1.5577
Chek1	56.2176	1.5462
Hic2	57.2177	1.5351
Atp8a1	461.7237	1.5229
Aifm1	295.0714	1.5058

Upregulated genes in Achilles tendon from *Dicer* cKO mouse by RNA-sequencing which are also predicted by TargetScan for target candidate genes of miR-135a. Differentially expressed genes (1.5-fold difference) are shown as the ratio of *Dicer* cKO to control mice.

## Discussion

The present study investigated tendon-specific *Dicer* cKO mice using *Scx* ^Cre/+^ KI mice. *Dicer* cKO mice exhibited immature tendons with impaired functions through the formation of abnormal collagen fibrils. In previous report, transcriptional factor *Scx* ^−/−^ mice exhibited severe disruption of force-transmitting tendons. However, ligaments, which are tissues connecting bone to bone and closely resemble tendons in their components, and short-range anchoring tendons were not affected (Murchison et al., 2007). Another recent study has been reported that *Scx* ^Cre/Cre^ KI mice (=*Scx* KO) also exhibit morphological defects in force-transmitting and intermuscular tendons, and ligament. However, *Scx* ^Cre/+^ KI mice (=*Scx*Ht) feature grossly/histologically normal tendons/ligaments (Yoshimoto et al., 2017). The present study demonstrates that hypoplastic tendons with abnormal collagen fibrils in *Dicer* cKO mice are significantly more present compared to *Scx*Ht mice. Thus, hypoplastic tendons in *Dicer* cKO mice could depend on the deletion of *Dicer*, rather than on heterogenic *Scx* in *Scx* ^Cre/+^ KI mice. In the present study, however, the expression level of *Dicer* and most miRNAs was not dramatically downregulated in tendons of *Dicer* cKO mice. This could be explained by the following reasons, as previously described (Yoshimoto et al., 2017); 1) Cre-mediated recombination in *Scx* ^Cre/+^ KI;*Dicer* ^flox/flox^ mouse has low efficiency and 2) The mosaic Cre-mediated recombination which occurred because of heterogeneity in endogenous *Scx* mRNA levels and time window of *Scx* expression in the *Scx*-expressing cells. Thus, the phenotype of tendon and ligament in *Dicer* cKO mice in this study might be milder. Indeed, generated *Dicer* cKO mice using *Scx*Cre Tg mice (Sugimoto et al., 2013) exhibited embryonic lethality or they immediately die after birth because of thoracic deformity (data not shown). Although previous reports demonstrate that expression levels of most miRNAs are reduced in *Dicer* and *Drosha* KO cells (Kobayashi et al., 2008; Kobayashi et al., 2015; Kim et al., 2016; Guo and Wang, 2019), in the present study, most miRNAs were not dramatically downregulated in tendons from *Dicer* cKO mice. Several studies also demonstrate that especially tissue specific- and tissue-enriched miRNAs stably exist in the tissues even after DICER inactivation (Pong and Gullerova, 2018; Oikawa et al., 2019a; Oikawa et al., 2019b; Vechetti et al., 2019). In Drosha, among 1,881 human miRNAs, only 311 miRNAs species are defined as “DROSHA dependent” (Kim et al., 2021). Thus, we may need to further understand miRNAs’ biogenesis.

Many knockout mice exhibiting collagen fibril abnormalities such as *Tnmd* and *Mkx* genes have been shown to have reduced biomechanical strength of the Achilles tendon (Docheva et al., 2005; Ito et al., 2010; Delgado Caceres et al., 2018). Although the fragility of tendon affects its strength remarkably, biomechanical factors, such as tensile strength, were not reduced in the Achilles tendon of *Dicer* cKO mice. Although the causes can be multifactorial, the elongation of Achilles tendon in *Dicer* cKO mice was increased and *Dicer* cKO mice had claudication. This result was demonstrated by our newly developed system which can easily evaluate function of animals by analyzing their movements using artificial intelligence (AI). This unique system has been trained to recognize anatomical landmarks in mice, allowing us to evaluate the gait function using only the video taken by a smartphone. The results of the present study showed that the *Dicer* cKO mice had an excessive range of ankle motion during gait. Previous study shows patients with chronic Achilles tendon injuries exhibit a greater ankle range of motion during running compared with normal subjects, which might support the assertion of our findings (Donoghue et al., 2008). This result suggests that *Dicer* cKO mice, having lost the stiffness in the Achilles tendon, could not control ankle motion during gait appropriately. In the future, such simple and inexpensive analysis of animal movements will be increasingly required to evaluate functions and pain in animal model.

Tendon fibroblasts, which are thought to be responsible for the maturation, maintenance and healing of tendon, are the main cell type in tendons. Approximately 30% of filtered transcripts were differentially regulated between tendons of a given species, and nearly 60% of the filtered transcripts present in anatomically similar tendons were different between species. This study indicates that tendon is a surprisingly heterogenous tissue with substantial genetic variation based on anatomical location and species (Disser et al., 2020). Recently, De Micheli et al. identified 11 distinct types of cells in mouse Achilles tendons, including three populations of tendon fibroblasts with *Col1a1* expressing (tendon fibroblasts 1, tendon fibroblasts 2 and Junctional fibroblasts) using single-cell transcriptomic analysis (De Micheli et al., 2020). Among top differentially expressed genes, ECM-binding genes *osteopontin* (*Spp1*) and *dermatopontin* (*Dpt*), are enriched in tendon fibroblasts 1 and 2, respectively (De Micheli et al., 2020). In the present study, *Dpt* and highly expressed tendon matrix genes in tendon fibroblasts such as *Col1a1* and *Fibromodulin* (*Fmod*), were significantly decreased in Achilles tendons of *Dicer* cKO mice compared with Control mice. Thus, these results indicate that the impaired gene expression patterns in *Dicer* downregulated-tendon fibroblasts were the cause of tendon hypoplasticity with abnormal collagen fibrils in *Dicer* cKO mice. The expression of these tendon matrix genes is regulated by transcriptional factors, such Scx, Mkx, and EGR1 (Murchison et al., 2007; Ito et al., 2010; Guerquin et al., 2013). EGR1 and Scx regulate transcription downstream of mechanical signaling during tendon fibroblast differentiation, tendon formation and healing by regulating tendon-related genes such as *Col1a1* (Lejard et al., 2011; Guerquin et al., 2013; Gaut et al., 2016; Gumucio et al., 2020). *Scx*, *Mkx*, and *EGR1* were downregulated in *Dicer* cKO mice compared with Control mice. Thus, although tendon hypoplasticity and impaired tendon healing in *Dicer* cKO mice might be caused by downregulated *Scx, Mkx* and *EGR1* in *Dicer*-deleted tendon fibroblasts, *EGR1* were downregulated in *both Dicer* cKO mice and *Scx* Ht mice compared with Control mice (Supplementary Figure S2A). Recently, *Tppp3*, and *Pdgfra* expressing cell population are identified as potential TSPCs and involved in tendon healing (Staverosky et al., 2009; Harvey et al., 2019; De Micheli et al., 2020). Thus, the impairment of tendon development and tendon healing in *Dicer* cKO mice might be associated with TSPCs because the expression of *Tppp3* and *Pdgfra* was significantly decreased in Achilles tendon of *Dicer* cKO mice. Furthermore, the function of miR-1247 was not reported in tendon. However, downregulated miR-135a in *Dicer* cKO mice was reported to be downregulated in aged compared with young TSPCs and overexpression of miR-135a in young TSPCs suppresses senescence, promotes proliferation, and induces migration and tenogenic differentiation (Chen et al., 2015). Thus, the number and/or function of TSPCs in tendon may be reduced in *Dicer* cKO mice. Furthermore, in the present study, miR-135a might play an important role in tendon maturation and healing because miR-135a mimic increased the expression of *Tnmd* and *Tppp3* in iATDF. Indeed, the upregulated genes, including the 21 target candidate genes for miR-135a, were involved in mitotic cell cycle based on the result of GO analysis (Supplementary Figures S2B,C). Thus, the upregulated genes in Scx-positive TSPCs including target genes that are directly regulated by miR-135a might cause immature tendon *via* abnormal TSPCs (proliferation and differentiation) in *Dicer* KO mice (Table 1 and Supplementary File).

DICER has a role in the biogenesis of most, if not all, miRNA and is essential for mammalian development, with *Dicer*-deficient mice dying due to a lack of detectable multipotent stem cells or differentiation capacity (Bernstein et al., 2003; Kanellopoulou, 2005; Kim et al., 2016). It has been reported that *Dicer* plays an important role in the development of the musculoskeletal system through the regulation of cell death, proliferation, and differentiation (Harfe et al., 2005; O’Rourke et al., 2007; Kobayashi et al., 2008; Gaur et al., 2010; Mizoguchi et al., 2010; Cheung et al., 2012; O’Toole et al., 2017). In the present study, it is unclear whether the fragility of tendon in *Dicer* cKO mice depends on Dicer-dependent impaired miRNA biogenesis or deficiency of essential DICER function. Although the approach that present study has taken may not be enough to identify tendon specific- or tendon functional-miRNAs, we focused on three differentially expressed and tendon-specifically expressed miRNAs between the tendons of Control and *Dicer* cKO mice from miRNA profiling using RNA sequencing. We should further examine the causes of tendon hypoplasia in *Dicer* cKO mice including the role of these miRNAs in tendon using gain- and loss of function mice, because it may not only be due to impaired miRNA biogenesis but also other functions of DICER such as DNA damage response (Burger et al., 2017). Recently, the peptide-mediated miRISC inhibition has been employed as an alternative approach for analyzing miRNA function without ablation of core miRNA biogenesis factors such as DICER (La Rocca et al., 2021). This approach may lead us to further interpretation in the role of miRNAs in various tissues including tendon.

Together, in this study, tendon-specific *Dicer* cKO mice exhibited immature tendons in which collagen fibrils have unordered structure with the downregulation of tendon-related genes and miRNAs. We identified miR-135a which were highly expressed in tendon and downregulated by *Dicer* deletion. Thus, DICER and miRNAs play an important role in tendon maturation, and miR-135a may have the potential to become key miRNA for tendon maturation and healing.

## Data Availability

The datasets presented in this study can be found in online repositories. The names of the repository/repositories and accession number(s) can be found below: NCBI's Gene Expression Omnibus (GEO) accession number GSE186353 (https://www.ncbi.nlm.nih.gov/geo/query/acc.cgi?acc=GSE186353).
