# Supplementary material for: Tendon-Specific Dicer Deficient Mice Exhibit Hypoplastic Tendon Through the Downregulation of Tendon-Related Genes and MicroRNAs
Source: Front Cell Dev Biol. 2022 Jun 14;10:898428. doi: 10.3389/fcell.2022.898428 (PMC9241168; doi:10.3389/fcell.2022.898428)
Supplement: Supplementary file 1 [file DataSheet1.PDF]

Supplementary Materials and Methods

Supplementary Methods

RNA sequencing analysis

Total RNA from Achilles tendon of Control, *Scx*Ht and *Dicer* cKO mice (one sample per group) was isolated using Isogen reagent (Nippon gene, Tokyo, Japan) and RNA purification kit (Direct-zol RNA microprep, Zymo Research, California, USA). RNA libraries were generated and sequenced by K. K. DNAFORM (Yokohama, Japan). The quality and quantity of the RNA samples were checked using an Agilent bio-analyzer. Paired end RNA-Seq libraries and small RNA-seq libraries were generated by K. K. DNAFORM using the Illumina provided protocol. Both libraries were sequenced on an Illumina HiSEQ4000 using Illumina provided reagents and protocols. Adaptor sequences were removed from sequenced reads, and the trimmed reads were mapped to the GRCm38-mm10 assembly of the mouse genome with Tophat and bowtie2. Raw read counts were then assigned to genes using HTSeq. Differential gene expression was analyzed with the R Bioconductor DESeq2 package (Love et al., 2014) using shrinkage estimation for dispersions and fold changes to improve stability and interpretability of estimates. MA plots were generated using custom R scripts. Differential expressed genes (baseMean>50 counts, fold change>1.67 or <0.60) were extracted and subsequently imported into gene ontology enrichment analysis with Metascape (https://metascape.org) (Zhou et al., 2019).

The RNA-seq and small RNA-seq data are available under GEO Series accession number GSE186353 (https://www.ncbi.nlm.nih.gov/geo/query/acc.cgi?acc=GSE186353).

REFERENCE

Love, M.I., Huber, W., and Anders, S. (2014). Moderated estimation of fold change and dispersion for RNA-seq data with DESeq2. *Genome Biology* 15(12)**,** 550. doi: 10.1186/s13059-014-0550-8.

Zhou, Y., Zhou, B., Pache, L., Chang, M., Khodabakhshi, A.H., Tanaseichuk, O., et al. (2019). Metascape provides a biologist-oriented resource for the analysis of systems-level datasets. *Nat Commun* 10(1)**,** 1523. doi: 10.1038/s41467-019-09234-6.

Supplementary Figures

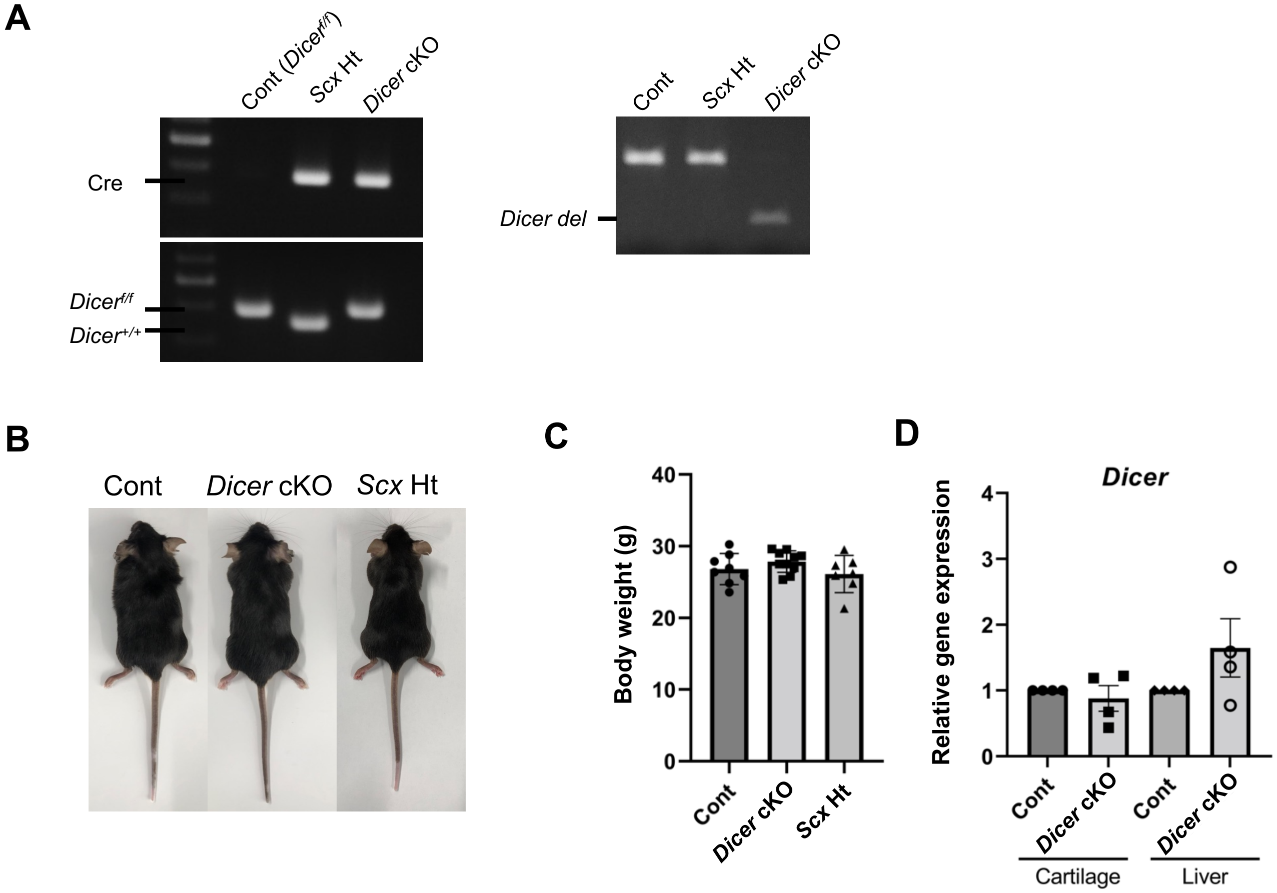

Supplementary Figure 1. (A) Genotyping of Control (*Dicer*$^{\text{flox/flox}}$)*, Scx*Ht (*Scx*$^{\text{Cre/+}}$*Dicer*$^{+/+}$) and *Dicer* conditional Knockout mice (*Scx*$^{\text{Cre/+}}$*Dicer*$^{\text{flox/flox}}$) by genomic DNA PCR from tail tip. **(B)** Growth was normal in mice of each genotype. **(C)** Body weight of Cont, *Dicer* cKO and *Scx*Ht mice at 10 weeks of age (Cont mice n=8, *Dicer* cKO mice n=10, *Scx*Ht mice n=7). Data are represented as mean ± S.D. Comparison of mean values was performed using one-way ANOVA and LSD test. **(D)** The expression of Dicer was not downregulated in cartilage and liver of Cont and *Dicer* cKO mice. Data are represented as mean ± SEM. Comparison of *Dicer* expression was performed using Welch's *t* test. n.s. : no significant difference. Cont: Control.

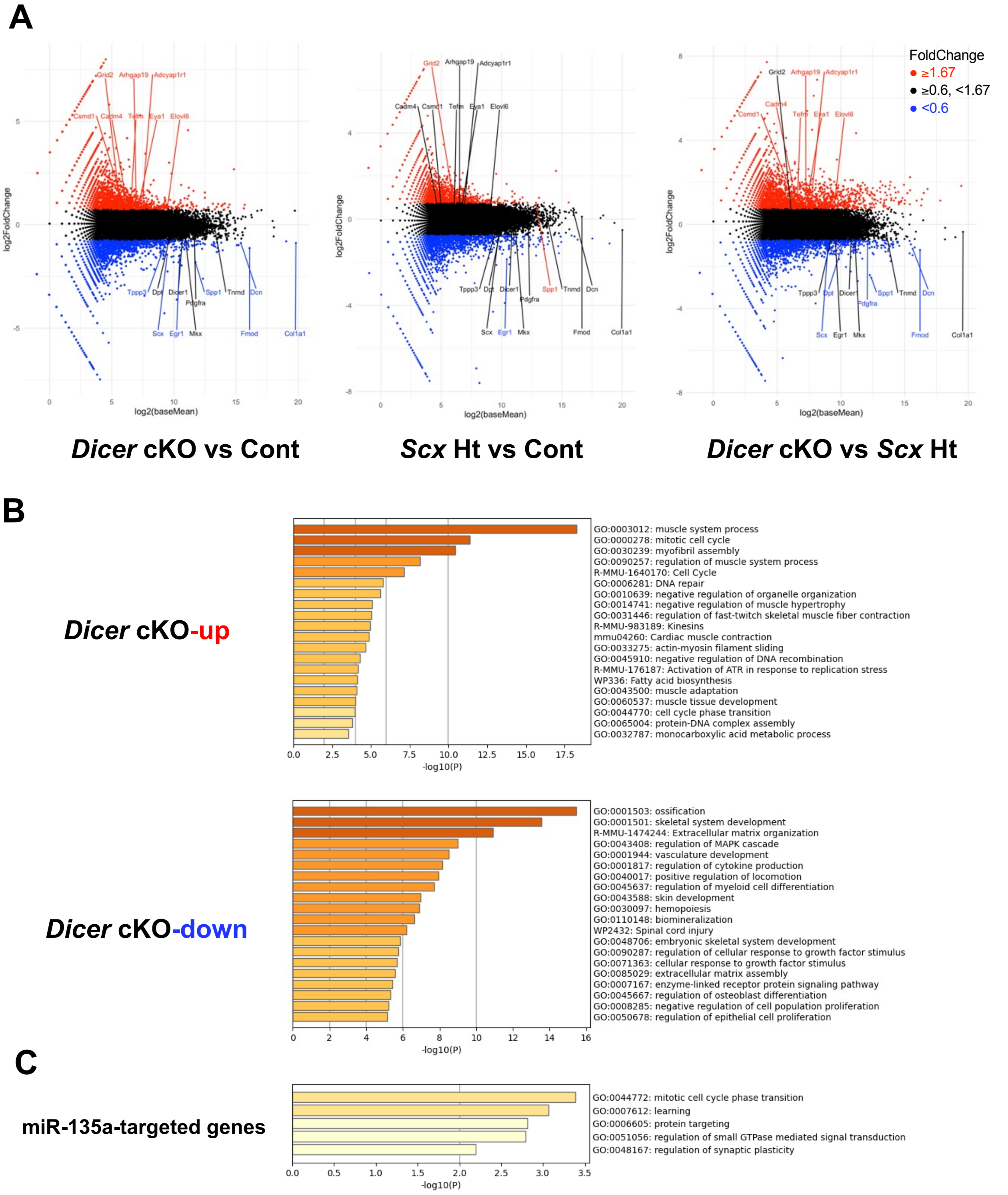

Supplementary Figure 2. (A) MA plots for pair comparison of the transcriptomes of Achilles tendon from Control, *Dicer* cKO or *Scx*Ht mice. Red color dots resembling fold change of sequence reads are greater than 1.67, blue color dots resembling fold change are less than 0.6; **(B)** Top 20 enriched gene ontology terms in up- or down-regulated gene sets of *Dicer* cKO mouse Achilles tendon; **(C)** Gene ontology enrichment analysis for the 21 target candidate genes for miR-135a in the Achilles tendon of *Dicer* cKO mouse.

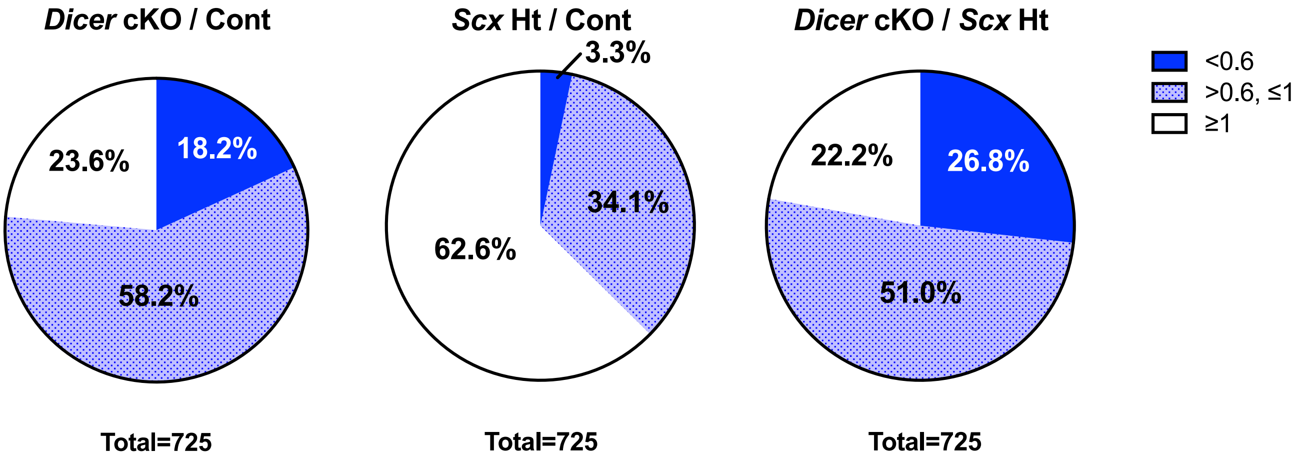

Supplementary Figure 3. Pie charts showing the percentage of tendon fibroblast-related genes with different degrees of downregulation compared to the overall tendon fibroblast-related genes based on the RNA-seq data. n=1 mouse for each genotype.

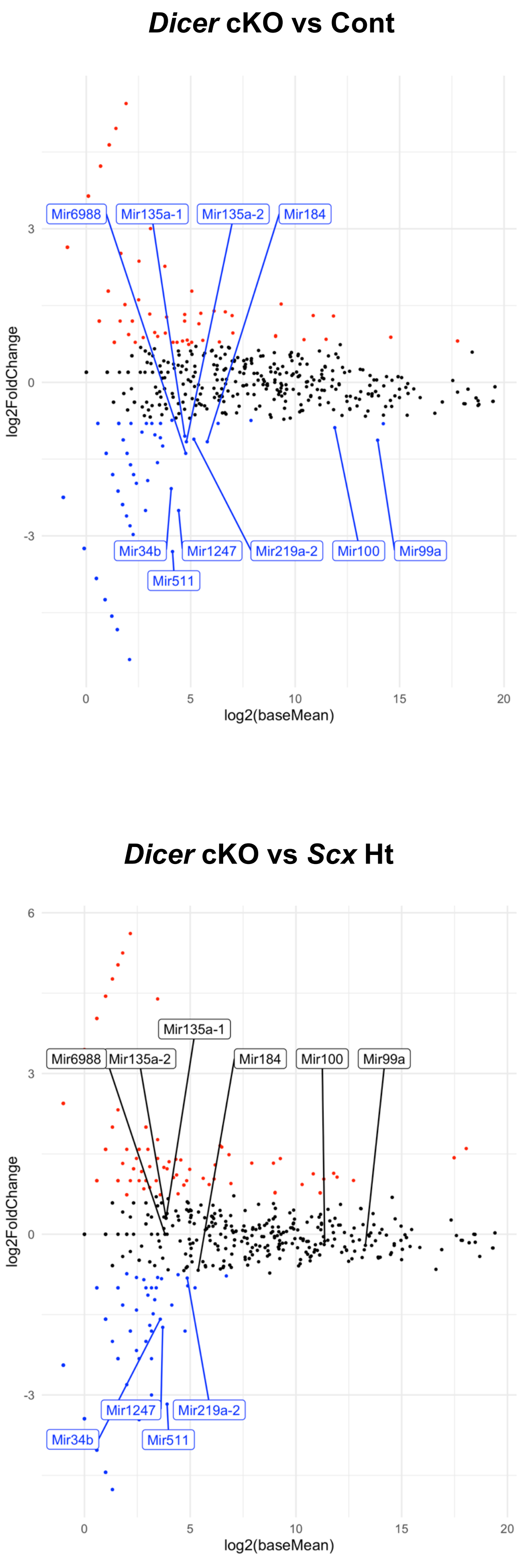

Supplementary Figure 4. MA plots for pair comparison of the small RNA transcriptomes of Achilles tendon from Cont, *Dicer* cKO or *Scx*Ht mice. Red color dots resembling fold change of sequence reads are greater than 1.67, blue color dots resembling fold change less than 0.6. n=1 mouse for each genotype.

Supplementary Table 1.

Gene Name	Assay ID
Dicer1	Mm00521731m1
Scx	Mm01205675m1
Mkx	Mm00617017m1
Egr1	Mm00656724m1
Col1a1	Mm00801666g1
Col3a1	Mm01254476m1
Tnmd	Mm00491594m1
Fmod	Mm00491215m1
Bgn	Mm00455918m1
Dcn	Mm00514535m1
Dpt	Mm00498111m1
Spp1	Mm00436767m1
Pdgfra	Mm0044070m1
Tppp3	Mm01251070m1
Gapdh	Mm99999915g1
has-miR-135a	RT/TM 002232
mmu-miR-511	RT/TM 002549
has-miR-1247	RT/TM 002893
U6 snRNA	RT/TM 001973

Real time PCR was performed with the TaqMan Gene Expression Assay and microRNA Assay probes.

Supplementary Table 2.

Rank	Gene Name	Expression (base mean)	*Dicer* cKO/Cont Ratio
1	miR-511	17.485	0.101
2	miR-1247	21.426	0.176
3	miR-34b	16.756	0.237
4	miR-6988	27.11	0.382
5	miR-135a-2	27.713	0.447
6	miR-184	55.427	0.447
7	miR-99a	15795.958	0.457
8	miR-219a-2	35.527	0.463
9	miR-135a-1	26.312	0.483
10	miR-100	3814.216	0.541

Supplementary Table 2. Top 10 of the most downregulated miRNAs in Achilles tendon from *Dicer* cKO mouse by smallRNA-sequencing

*miR-6988 were registered in mouse only.

Supplementary File. List of up- and down-regulated genes in Achilles tendon from *Dicer* cKO mouse by RNA-sequencing (cut off: *Dicer* cKO/Control ratio >1.5, baseMean >50 counts).

Supplementary Videos. The movement of the shaved hindlimb of mice was recorded over 30 images per second of video, and the part of the video where the mice were continuously positioned in the center two-thirds of the image was analyzed. Residual Network (ResNet-50), one of the neural networks pre-trained by ImageNet was used to learn transitions in the mice's gait videos and automatically track the mice's anatomical landmarks: the knee joint, heel, and fifth metatarsal head. The coordinates of the three obtained landmarks were obtained, and the angles between the three landmarks were calculated using inverse trigonometric functions. The angular velocity was calculated by time derivative of the obtained change in the ankle joint angle.
